# Exploring the Relationship between Rising Temperatures and the Number of Climate-Related Natural Disasters in China

**DOI:** 10.3390/ijerph18020745

**Published:** 2021-01-16

**Authors:** Mingan Zhu, Bihang Fan

**Affiliations:** 1 State Key Laboratory of Hydraulics and Mountain River Engineering, College of Water Resource and Hydropower, Sichuan University, Chengdu 610065, China; zhumingan02@gmail.com; 2 School of Geography, University of Leeds, Leeds LS2 9JT, UK

**Keywords:** climate change, disaster mitigation, elasticity coefficient, time series, varying patterns, wavelet analysis

## Abstract

Warming has strongly influenced the quantity and variability of natural disasters around the globe. This study aims to characterize the varying patterns between rising temperatures and climate-related natural disasters in China from 1951 to 2010. We examined the overall trend in the patterns of an 11-year cycle, and climate-related natural disaster responses to periods of rising and dropping temperature. We used Morlet wavelet analysis to determine the length of a temperature cycle period, and the arc elasticity coefficient to assess the number of climate-related natural disasters in response to the changing temperature. We found that: (1) the overall relationship between temperature and the number of climate-related natural disasters was positive; (2) however, on the cycle level, the pattern of climate-related natural disasters was found to be independent of temperature variation; (3) on the rise-drop level, temperature increases were associated with declines in the number of climate-related natural disasters. Moreover, as temperature decreased, the number of climate-related natural disasters increased substantially, such that temperature had a more considerable influence on the quantity of climate-related natural disasters during the temperature-drop period. Findings in this study can help enhance the dissemination of warning and mitigation efforts to combat natural disasters in the changing climate.

## 1. Introduction

Climate change occurs due to an imbalance between outgoing and incoming radiation in the Earth’s atmosphere [1]. Largely as a result of the combustion of fossil fuels, greenhouse gas concentrations have reached record levels in the atmosphere, causing a remarkable increase in global temperature. In recent decades, researchers have identified an increasing number of climate-related natural disasters, such as droughts, floods, heatwaves, storms, wildfires, landslides, and epidemics. As a result of global warming, the Earth’s hydrologic cycle has shifted as warmer air retains more moisture than cooler air [1]. For example, Trenberth et al. [1] indicated that droughts are becoming more extensive due to the increased evapotranspiration associated with the extra heat added to the climate system. Extreme temperatures (such as heat waves) and floods have also become more frequent, more intense, and more prolonged as temperatures rise, in addition to the acceleration of melting snowpack (and glaciers) and more numerous heavy rainfalls [2,3]. The observed increases in the frequency of storms [4], wildfires [5], slope failures (e.g., landslides and mudflows) [6], infectious diseases [7], outbreaks of insects vectors of disease [8], and insect infestation [9] are closely related to the warming climate.

Recent studies have even identified a correlation between the rise in temperature and seismic activity [10,11]. Global warming is associated with periods of drought and heavy precipitation, which can cause entire mountain ranges to rise and fall at the scale of tens of millimeters [12], and with glacial retreat, which can trigger seismic activity as the crust isostatically rebounds [13,14]. Such stress changes could potentially trigger earthquakes on nearby faultlines. Another warming-related process that is related to seismic activity is deglaciation [13]. Therefore, Che et al. [14] suggested that special attention should be paid to the relationship between earthquakes and the rising soil temperature in the warm permafrost regions on the Tibet Plateau, a primary active seismic zone in China. Taken together, climate-related natural disasters have severely impacted human livelihoods and natural environments around the world [15,16].

The average global surface temperature increased by approximately 0.7 °C during the 20th century, but it is predicted to keep rising by another 1.8 to 5.8 °C by the end of this century [17]. Most regional climate studies, such as in South Asia [18], central-western Europe [19], central and eastern Europe [20], Tibetan Plateau and other parts of China [21], western central Africa [22], and Australia [23], have yielded findings consistent with the increasing temperature. Therefore, to prepare the warning dissemination and mitigation strategy for future disasters, it is crucial to enhance an in-depth understanding of the relationship between climate-related natural disasters and rising temperatures [17,24].

As the planet warms, the risks and implications are expected to compound. Existing research has revealed such a relationship from different aspects. Bergholt and Lujala [25] suggested that under climate warming, more frequent and severe climate-related disasters will significantly impede GDP growth. Slettebak [26] confirmed that increasingly frequent climate-related natural disasters can potentially explain the outbreak of civil conflicts over the past seven decades. Keim [27] and Popovski and Mundy [28] demonstrated that global warming could increase the number and severity of climate-related natural disasters and have a negative effect on public health. The impacts of climate-related natural disasters due to warming seem poised to be far-reaching, requiring more studies to examine the potential risks of the increasing frequency of climate-related natural disasters to social and environmental systems [29].

In order to effectively warn populations and prepare mitigation strategies for future disasters, it is crucial that we enhance our in-depth understanding of the relationship between rising temperatures and natural disasters. However, the current literature on the relationship between rising temperature and the incidence of natural disasters remains incomplete and sometimes conflicting. Although the long-term trends in the annual frequency of climate-related natural disasters have been successfully attributed to warming in numerous studies, e.g., [2,3,4,5,6,7,8,9,10,11], patterns in the response of climate-related natural disasters to climate change are inadequately explored [16]. Uncovering these various patterns is challenging. For example, air temperature has not risen steadily, but rather with fluctuations; long-term trends ignore patterns within decade-long time intervals. Such variable patterns should be investigated at multiple, carefully chosen time intervals to reflect the nature of warming cycles.

This study aims to explore the varying patterns of the quantitative relationship between global warming and natural disasters in China, with an approach suitable to account for climate change, e.g., [30,31,32]. Taking into account the multiple scales of the patterns of note, this study used Wavelet analysis to divide the temperature time series into periods. Then, within each period, we extracted both the increasing and decreasing components from the temperature time series. The relationship between temperature and climate-related natural disasters were then examined at all three time scales: the entire study period, the temperature cycling periods, and the fluctuations within each temperature cycling period. This study afforded particular focus on the most granular of these relationships, i.e., the rise-drop fluctuations, to describe the fundamental relationship between warming and the change in climate-related natural disasters to complement existing knowledge. The results reported here should help researchers and policy-makers to predict trends and variability in natural disaster occurrence as our climate continues to change.

## 2. Materials and Methods

### 2.1. Data

The data for climate-related natural disasters (1951 to 2010) were downloaded from the Emergency Disasters Database (EM-DAT) of the Centre for Research on the Epidemiology of Disasters (CRED). EM-DAT is a carefully curated global disaster dataset with annual records starting from 1900, including human-induced and natural disasters. We isolated nine types of climate-related natural disasters that have previously been associated with a warming climate: drought, earthquake, epidemic, extreme temperature, flood, insect infestation, mass movement (e.g., slope failures), storm, and wildfires. To ensure a sufficient sample size, we considered climate-related natural disasters as a whole. Because the data of climate-related natural disasters prior to 1980 were sometimes not fully recorded, the study placed more emphasis on the period from 1981 to 2010. Daily surface air temperature data (1951 to 2010) in China were taken from the National Climatic Data Center of the China Meteorological Administration (CMA) and were used to compute the annual mean temperature.

### 2.2. Wavelet Analysis

Wavelet analysis originated as a mathematical method for time-frequency or time-scale analysis in the early 1980s [33,34]. By analyzing the characteristics of localized variations within a time series, one can quantify how the modes vary. Wavelet analysis has been successfully applied in numerous geophysical studies, including tropical convection [35], the El Niño-Southern Oscillation [36,37], atmospheric cold fronts [38], and temperature changes in central England [39]. A wavelet transform is the basic principle of Wavelet analysis: a one-dimensional signal spreading in two directions in time and frequency [33]. Thus, a detailed analysis of the climate system in a time-frequency structure can be achieved. The wavelet transformation for any function and its smooth function can be obtained via the convolution operation [34]. Then, by solving the second-order derivatives with respect to time, any inflection points are the values at which the function crosses the x-axis, i.e., the zero crossings [33,34]. This point may be regarded as a possible catastrophe point in the division of temperature cycles [39]. Once extracted from the time series, catastrophe points can be used to reveal the cycle information in the temperature time series.

We used the one-dimensional continuous wavelet transform (i.e., the Morlet wavelet method with a 1:50 scale) on the 5-year moving average temperature (5-YMAT) time series to determine the cycle length. We then performed a convolution operation on the 5-YMAT to get the harmonic of the period with the obtained cycle length. By comparing the specific oscillation of the harmonic with the 5-YMAT, we divided the time series into different cycles and further split them into rising and falling components within each cycle.

### 2.3. Elasticity Coefficient

Elasticity describes the response of one variable to the change of another variable. The best indicator of elasticity is the relative magnitude of the changes in two variables, i.e., the elasticity coefficient (*E*). This coefficient is often used in economic studies to reflect the dependence of the growth of one economic variable on the growth of another:

(1) $$E=\frac{\Delta Y/Y}{\Delta X/X}=\frac{\Delta Y}{\Delta X}\times \frac{X}{Y},$$

where Δ*X* and Δ*Y* indicate the change in variable *X* and variable *Y*, respectively.

When there is a need to measure the average elasticity of an arc between two points on the demand curve, the arc elasticity coefficient (*E_dr_*) is calculated as [40]:

(2) $$E_{dr}=\frac{\left(X_{2}{-X}_{1}\right)/\frac{\left(X_{2}{+X}_{1}\right)}{2}}{\left(Y_{2}{-Y}_{1}\right)/\frac{\left(Y_{2}{+Y}_{1}\right)}{2}},$$

In this study, the time series was treated in a manner similar to a demand curve. Data in the 5-YMAT were treated as the domain (*X*-value), and the corresponding annual number of climate-related natural disasters (ACND) was treated as the range (*Y*-value). The term *E_dr_* was used to reflect the change in ACND in response to the 5-YMAT. If *E_dr_* was negative, this indicated a negative correlation between temperature and natural disasters [40]. The value of |*E_dr_*| has an inverse relationship with the degree of change in variable *Y* caused by a unit change in variable *X*, such that a smaller value of |*E_dr_*| refers to a stronger response of *Y* to *X* [40]. In order to examine the magnitude of the relationship between 5-YMAT and ACND, we compared the |*E_dr_*| values between the rise and drop periods.

## 3. Results

### 3.1. Overall Patterns

**Figure 1:**
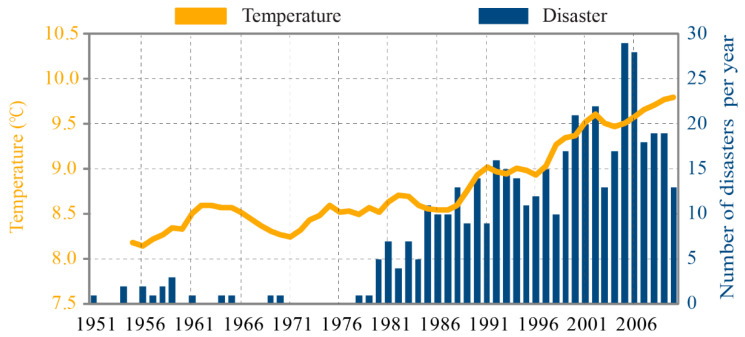
Time series of the 5-YMAT (5-year moving average temperature; yellow lines) and ACND (annual number of climate-related natural disasters; blue bars) from 1951 to 2010.

We identified a total of 664 climate-related natural disasters during the study period (Table A1). The overall pattern for temperature dynamics and year-to-year variations in ACND from 1951 to 2010 in China is shown in Figure 1. Over the entire study period, there was a positive and statistically significant relationship (*p* < 0.05) between the 5-YMAT and ACND. The 5-YMAT experienced fluctuating increases, rising from 8.1–8.5 °C in the 1950s to >9.5 °C after 2005. The ACND also increased significantly, peaking in 2005 (at 29 events). According to the overall patterns (Figure 1), we can interpret a clear pattern in the number of climate-related natural disasters associated with warming. In addition to the relationship between long-term trends, Figure 1 also depicts short-term fluctuations in both 5-YMAT and ACND. Thus, detailed analysis at these short periods can better reveal the response of climate-related natural disasters to temperature changes.

### 3.2. Short-Term Cyclical Patterns

**Figure 2:**
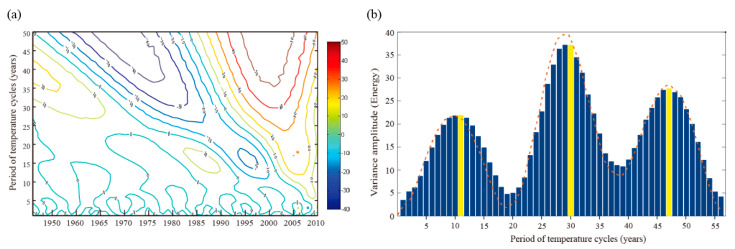
Results of the Morlet continuous wavelet transform analysis (**a**) and variances analysis (**b**). The yellow bars in (**b**) indicate the possible period of temperature cycles.

**Figure 3:**
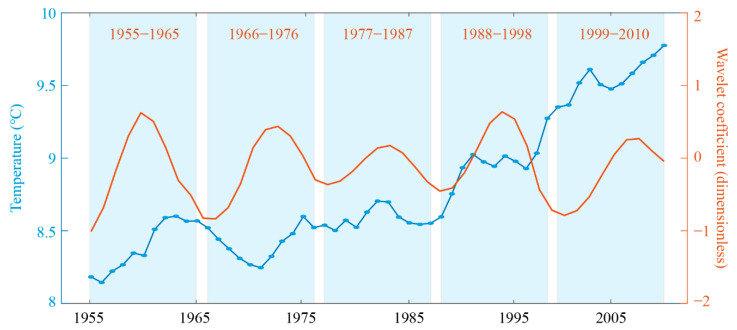
A comparison between the 5-YMAT (blue dotted curve) and the harmonic of the period of 11 years (orange curve). Blue areas indicate the division of the temperature time series into five cycles.

The results of the Morlet wavelet analysis are shown in Figure 2a, which identified multiple oscillation periods in the temperature time series, including 10–15 year and 25–30 year periods. Figure 2b depicts the result of the variance analysis of the Morlet wavelet transformation, indicating that the most likely positions of the perturbation energy were at 11, 30, and 47 years, so these intervals were taken as the inter-decadal cycle lengths for the temperature time series. To ensure that at least two full cycles were included in the study period, we focused on temperature cycles with an 11-year period. We then compared the 5-YMAT and the harmonic of the 11-year period (Figure 3), which indicates five cycles: 1955–1965, 1966–1976, 1977–1987, 1988–1998, and 1999–2000. Each cycle was further divided into a temperature-rise period and a temperature-drop period (Table 1). To facilitate the comparison with 5-YMAT, we used the five-year moving average ACND instead of the original ACND (also named ACND) and started it in the same year.

**Figure 4:**
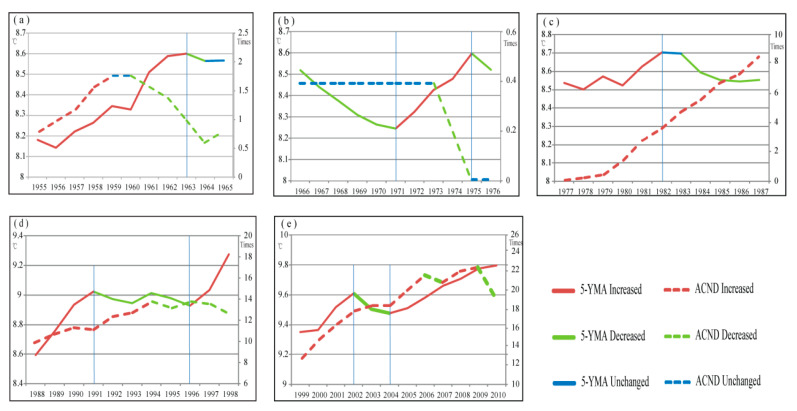
A comparison between the 5-YMAT and ACND in each cycle period in the temperature time series. (**a**) 1955–1965, (**b**) 1966–1976, (**c**) 1977–1987, (**d**) 1988–1998, and (**e**) 1999–2010.

A comparison of the changes in the 5-YMAT and ACND in each cycle period is shown in Figure 4. At this time scale, we identified no significant correlation between the two variables in each cycle period. For example, Figure 4a shows an increasing 5-YMAT with ACND that grew at first but then decreased. As 5-YMAT increased in any period, ACND could decline, increase, or increase and then decline, whereas when 5-YMAT fell, ACND could remain unchanged, decrease, or even increase. We captured no general pattern of climate-related natural disaster response to temperature changes within the identified cycle periods.

### 3.3. Rise-Drop Sensitivity

**Figure 5:**
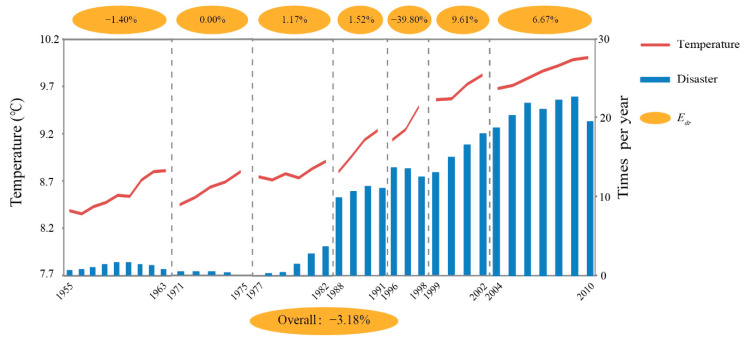
The arc elasticity coefficient (*E_dr_*) of 5-YMAT and ACND during temperature-rise periods. The coefficient values for each temperature cycle period are shown in the ovals above the figure. The corresponding coefficient for the entire temperature-rise period is shown in the oval below the figure.

Figure 5 shows the *E_dr_* values of 5-YMAT and ACND in the seven temperature-rise periods from 1955 to 2010. The value of *E_dr_* was negative in two periods (1955–1963 and 1996–1998), but it was positive across other rising periods. The overall *E_dr_* between 5-YMAT and ACND during the entire temperature-rise period was −3.18%, indicating that the number of climate-related natural disasters decreased as the temperature rose at the study scale.

**Figure 6:**
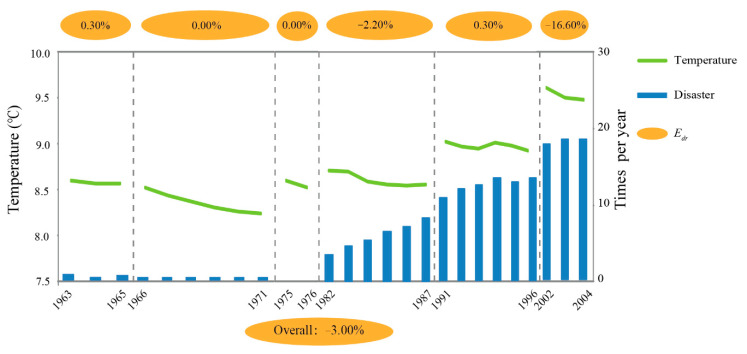
The arc elasticity coefficient (*E_dr_*) of 5-YMAT and ACND during temperature-drop periods. The coefficient values for each temperature cycle period are shown in the ovals above the figure. The corresponding coefficient for the entire temperature-rise period is shown in the oval below the figure.

Figure 6 depicts the *E_dr_* values of 5-YMAT and ACND during the six temperature-drop periods from 1955 to 2010. The value of *E_dr_* was negative in two periods (1982 to 1987 and 2002 to 2004), and the rest were slightly positive. The overall *E_dr_* between 5-YMAT and ACND throughout the entire temperature-drop period was 3.00%, indicating that ACND increased as the 5-YMAT declined at the study scale.

**Figure 7:**
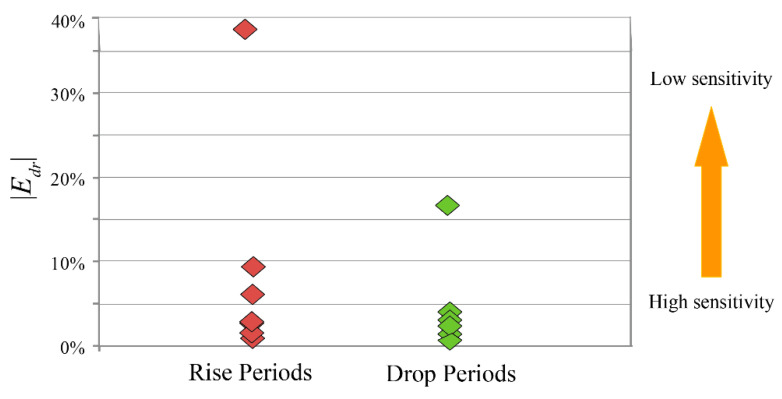
A comparison of the absolute value of the arc elasticity coefficient (|*E_dr_*|) between the temperature-rise period and temperature-drop periods.

Figure 7 contains the results of the absolute value calculations for Edr, where a smaller value of |*E_dr_*| indicates a more severe response of ACND to 5-YMAT. During the temperature-rise periods, |*E_dr_*| was mainly below 10%, with a maximum value of 39.80%. During the temperature-drop periods, |*E_dr_*| was mainly below 5%, with a maximum value of just 16.6%. This suggests that the number of climate-related natural disasters was more responsive to temperature when temperature was declining.

## 4. Discussion

Connecting climate trends to natural disasters has become an essential issue of significant scientific and public interest [16,41]. Many previous studies have provided evidence that the increasing number of natural disasters is closely associated with rising temperatures [24,25,26,27]. For example, the increased heat due to global warming that is added to the climate system and on the land surface induces more severe droughts and more frequent extreme temperature events [1,2,19,20,21]. Due to the changes in the hydrologic processes under warming, such as deglaciation, the melting of permafrost, extended drought periods, and more frequent heavy rainfalls, floods, landslides, wildfires, and earthquakes are expected [2,3,4,5,6,10,11,12,13,14]. Furthermore, a warmer climate intensifies infectious disease activity and infectivity, having an enormous effect on the distribution of many infectious diseases and insect infestation [7,8,9]. We confirmed the positive relationship between 5-YMAT and ACND across our entire study period (Figure 1). This suggests that unmitigated global warming is likely to expose people to larger numbers and/or more severe climate-related natural disasters in the future.

However, fluctuations in temperatures prevent a simple application of this broad relationship when explaining or predicting climate-related natural disasters in inter-decadal scales or shorter scales. At the cycle scale, the changes in ACND were found to be independent of temperature changes (Figure 5 and Figure 6), while the relationship in both the temperature-rise periods and temperature-drop periods were apparently negative. These short-term responses contradict the overall relationship between 5-YMAT and ACND (Figure 1). This result complements our understanding of the relationship between climate-related natural disasters and the change in temperature, e.g., [1,2,3,4,5,6,7,8,9,10,11,12,13,14], which indicates the necessity of examining varying patterns of natural disasters and changing climate at different temporal scales. It is often assumed, based on the broad pattern, that periods of warming should lead to higher risks of natural disasters and increased economic loss [25,26,27,28,29]. However, our results suggest that the potential impacts of climate-related natural disasters should not be neglected even during the temperature-drop periods. This is consistent with the findings of [15], which found that a decrease in spring temperature can intensify subsequent outbreaks of dust storms in northern China. Also, Zhang et al. [41] indicated that, despite the warming trend during the past 50 years, severe weather occurrences in China significantly decreased from 1961 to 2010. They further demonstrated that the reduction in severe weather days could be attributed to the weakening of the East Asian summer monsoon [41].

Although both temperature-rise periods and temperature-drop conditions were each associated with noticeable increases in ACND, we found that the number of disasters responded more strongly to periods of declining temperature (Figure 7). This reinforces the notion that a narrow focus on temperature-rise periods is misplaced. Therefore, in the context of an ongoing general warming trend, we advocate paying more attention to the risk of climate-related natural disasters even when temperature decreases.

The EM-DAT global disaster dataset used in this study is a free database that contains worldwide records of the occurrence and impact of natural and technological disasters from 1990 at the country level. This database opens new opportunities to link dynamics in natural disasters and the associated economic loss and mortality to ongoing climatic change e.g., [42,43]. However, the EM-DAT dataset consists of information from various sources, and the consistency of the data may be questionable. In our case, the selected types of disasters were likely underreported before 1980 (Figure 1). An updated disaster database with more complete records would strengthen our ability to describe and model the changes in climate-related natural disasters with global warming.

In addition to the Wavelet analysis method, there are other possible approaches, such as Empirical Mode Decomposition (EMD) and Ensemble Empirical Mode Decomposition (EEMD), to examine the cycle length of the temperature time series, e.g., [44]. Nonetheless, previous studies have also suggested 11 years as a reasonable cycle period for global or regional temperature time series [45], which is likely associated with the known solar activity period [46]. As the data used in this study are from China, a developing country, future studies should look at *E_dr_* in developed countries to understand the impact of industrialization on the relationship between warming and climate-related natural disasters. Finally, more research is required to pinpoint the physical mechanisms by which changing temperatures influence climate-related disasters. Such knowledge could be used to improve disaster modeling under global warming.

## 5. Conclusions

This study presents preliminary results of the varying patterns between temperature change and the incidence of climate-related natural disasters. We examined the overall trends and the response at the cycle level and the temperature rise-drop level. We found that analysis of varying patterns is a promising approach for predicting the trends and variability of natural disasters in future climate scenarios. Our study demonstrated a method to investigate how climate-related natural disasters will change with warming at different temporal scales. The results suggested that the short-term relationships between climate change and climate-related natural disasters are complex and that periods of cooling cannot be taken as a sign of reduced risk. This information can help enhance the warning dissemination and mitigation efforts to combat climate-related natural disasters in China.

## Figures and Tables

**Table 1:** A list of the cycle periods in temperature time series with the rise and drop period (and duration) of each.

Periods	Rise Periods (Duration in Years)	Drop Periods (Duration in Years)
1955–1965	1955–1963 (8)	1963–1965 (3)
1966–1976	1971–1975 (4)	1966–1971, 1975–1976 (7)
1977–1987	1977–1982 (5)	1982–1987 (6)
1988–1998	1988–1991, 1996–1998 (5)	1991–1996 (6)
1999–2010	1999–2002, 2004–2010 (8)	2002–2004 (3)

## Data Availability

The data presented in this study are available on request from the corresponding author.

## Appendix A

**Table A1:** The types and counts of climate-related natural disasters used for analysis in this study. We also included literature that supports the close relationship between each type of natural disaster and global warming.

Type of Disasters	Counts	Reference
Drought	32	[1]
Earthquake (seismic activity)	126	[10,11,12,13,14]
Epidemic	10	[7,8]
Extreme temperature	11	[2,19,20,21]
Flood	206	[3]
Insect infestation	1	[9]
Mass movement	61	[6]
Storm	211	[4,15]
Wildfire	6	[5]

## References

[1] Trenberth K.E., Dai A., van der Schrier G., Jones P.D., Barichivich J., Briffa K.R., Sheffield J. (2014). Global warming and changes in drought. Nat. Clim. Chang..

[2] Baldwin J.W., Dessy J.B., Vecchi G.A., Oppenheimer M. (2019). Temporally Compound Heat Wave Events and Global Warming: An Emerging Hazard. Earths Future.

[3] Huang X., Hall A.D., Berg N. (2018). Anthropogenic Warming Impacts on Today’s Sierra Nevada Snowpack and Flood Risk. Geophys. Res. Lett..

[4] Brooks H.E. (2013). Severe thunderstorms and climate change. Atmos. Res..

[5] Calder W.N., Parker D., Stopka C.J., Jiménez-Moreno G., Shuman B.N. (2015). Medieval warming initiated exceptionally large wildfire outbreaks in the Rocky Mountains. Proc. Natl. Acad. Sci. USA.

[6] Stoffel M., Huggel C. (2012). Effects of climate change on mass movements in mountain environments. Prog. Phys. Geogr. Earth Environ..

[7] Shuman E.K. (2010). Global Climate Change and Infectious Diseases. N. Engl. J. Med..

[8] Chaves L.F., Scott T.W., Morrison A.C., Takada T. (2014). Hot temperatures can force delayed mosquito outbreaks via sequential changes in Aedes aegypti demographic parameters in autocorrelated environments. Acta Trop..

[9] Dale A.G., Frank S.D. (2017). Warming and drought combine to increase pest insect fitness on urban trees. PLoS ONE.

[10] Viterito A. (2016). The Correlation of Seismic Activity and Recent Global Warming. J. Earth Sci. Clim. Chang..

[11] Masih A. An enhanced seismic activity observed due to climate change: Preliminary results from Alaska. Proceedings of the 8th International Conference on Environment Science and Engineering (ICESE2018).

[12] Argus D.F., Landerer F.W., Wiese D.N., Martens H.R., Fu Y., Famiglietti J.S., Thomas B.F., Farr T.G., Moore A.W., Watkins M.M. (2017). Sustained Water Loss in California’s Mountain Ranges During Severe Drought From 2012 to 2015 Inferred From GPS. J. Geophys. Res. Solid Earth.

[13] Swindles G.T., Watson E.J., Savov I.P., Lawson I.T., Schmidt A., Hooper A., Cooper C., Connor C.B., Gloor M., Carrivick J. (2018). Climatic control on Icelandic volcanic activity during the mid-Holocene. Geology.

[14] Che A., Wu Z., Wang P. (2014). Stability of pile foundations base on warming effects on the permafrost under earth quake motions. Soils Found..

[15] Fan B., Guo L., Li N., Chen J., Lin H., Zhang X., Shen M., Rao Y., Wang C., Ma L. (2014). Earlier vegetation green-up has reduced spring dust storms. Sci. Rep..

[16] Guo L., Fan B., Zhang F., Jin Z., Lin H. (2018). The clustering of severe dust storm occurrence in China from 1958 to 2007. J. Geophys. Res..

[17] Solomon S., Dahe Q., Martin M., Melinda M., Kristen A., Melinda M.B.T., Miller H.L., Zhenlin C. (2007). Climate Change 2007: The Physical Science Basis: Contribution of Working Group I to the Fourth Assessment Report of the Intergovernmental Panel on Climate Change.

[18] Naylor R.L., Battisti D.S., Vimont D.J., Falcon W.P., Burke M.B. (2007). Assessing Risks of Climate Variability and Climate Change for Indonesian Rice Agriculture. Proc. Natl. Acad. Sci. USA.

[19] Della-Marta P.M., Haylock M.R., Luterbacher J., Wanner H. (2007). Doubled Length of Western European Summer Heat Waves since 1880. J. Geophys. Res. Atmos..

[20] Bartholy J., Pongracz R. (2007). Regional Analysis of Extreme Temperature and Precipitation Indices for the Carpathian Basin from 1946 to 2001. Glob. Planet. Chang..

[21] You Q., Kang S., Aguilar E., Yan Y. (2008). Changes in Daily Climate Extremes in the Eastern and Central Tibetan Plateau during 1961–2005. J. Geophys. Res..

[22] Aguilar E., Aziz Barry A., Brunet M., Ekang L., Fernandes A., Massoukina M., Mbah J., Mhanda A., do Nascimento D.J., Peterson T.C. (2009). Changes in Temperature and Precipitation Extremes in Western Central Africa, Guinea Conakry, and Zimbabwe, 1955–2006. J. Geophys. Res..

[23] Alexander L.V., Arblaster J.M. (2009). Assessing Trends in Observed and Modelled Climate Extremes over Australia in Relation to Future Projections. Int. J. Clim..

[24] Birkmann J., von Teichman K. (2010). Integrating Disaster Risk Reduction and Climate Change Adaptation: Key Challenges—Scales, Knowledge, and Norms. Sustain. Sci..

[25] Bergholt D., Lujala P. (2012). Climate-Related Natural Disasters, Economic Growth, and Armed Civil Conflict. J. Peace Res..

[26] Slettebak R.T. (2012). Don’t Blame the Weather! Climate-Related Natural Disasters and Civil Conflict. J. Peace Res..

[27] Keim M.E. (2011). Preventing Disasters: Public Health Vulnerability Reduction as a Sustainable Adaptation to Climate Change. Disaster Med. Public Health Prep..

[28] Popovski V., Mundy K.G. (2012). Defining Climate-Change Victims. Sustain. Sci..

[29] Semenza J.C., Ploubidis G.B., George L.A. (2011). Climate Change and Climate Variability: Personal Motivation for Adaptation and Mitigation. Environ. Health.

[30] Otero R.C., Marti R.Z. (1995). The Impacts of Natural Disasters on Developing Economies: Implications for the International Development and Disaster Community.

[31] Benson C., Clay E.J. (2004). Understanding the Economic and Financial Impacts of Natural Disasters.

[32] Benson C., Clay E.J. (2003). Disasters, Vulnerability and the Global Economy. Build. Safer Cities Future Disaster Risk.

[33] Morlet J., Arens G., Fourgeau E., Glard D. (1982). Wave Propagation and Sampling Theory—Part I: Complex Signal and Scattering in Multilayered Media. Geophys..

[34] Morlet J., Arens G., Fourgeau E., Giard D. (1982). Wave Propagation and Sampling Theory—Part II: Sampling Theory and Complex Waves. Geophysics.

[35] Weng H., Lau K.M. (1994). Wavelets, Period Doubling, and Time–Frequency Localization with Application to Organization of Convection over the Tropical Western Pacific. J. Atmosph. Sci..

[36] Gu D., Philander S.G.H. (1995). Secular Changes of Annual and Interannual Variability in the Tropics during the Past Century. J. Clim..

[37] Wang B., Wang Y. (1996). Temporal Structure of the Southern Oscillation as Revealed by Waveform and Wavelet Analysis. J. Clim..

[38] Gamage N., Blumen W. (1993). Comparative Analysis of Low-Level Cold Fronts: Wavelet, Fourier, and Empirical Orthogonal Function Decompositions. Mon. Wea. Rev..

[39] Baliunas S., Frick P., Sokoloff D., Soon W. (1997). Time Scales and Trends in the Central England Temperature Data (1659–1990): A Wavelet Analysis. Geophys. Res. Lett..

[40] Casler S. (2013). Cost Minimization and Elasticity Estimation: A Two-Input, Two-Time Period Analysis. Econ. Instr..

[41] Zhang Q., Ni X., Zhang F. (2017). Decreasing trend in severe weather occurrence over China during the past 50 years. Sci. Rep..

[42] Delbiso T.D., Altare C., Rodriguez-Llanes J.M., Doocy S., Guha-Sapir D. (2017). Drought and child mortality: A meta-analysis of small-scale surveys from Ethiopia. Sci. Rep..

[43] Mehrabi Z., Donner S., Rios P., Guha-Sapir D., Rowhani P., Kandlikar M., Ramankutty N. (2019). Can we sustain success in reducing deaths to extreme weather in a hotter world?. World Dev. Perspect..

[44] Ji F., Wu Z., Huang J., Chassignet E.P. (2014). Evolution of land surface air temperature trend. Nat. Clim. Chang..

[45] Sunkara S.L., Tiwari R.K., Sunkara S.L., Tiwari R.K. (2016). Wavelet analysis of the singular spectral reconstructed time series to study the imprints of solar-ENSO-geomagnetic activity on Indian climate. Nonlinear Process. Geophys..

[46] Li Z., Yue J., Xiang Y., Chen J., Bian Y., Chen H. (2018). Multiresolution Analysis of the Relationship of Solar Activity, Global Temperatures, and Global Warming. Adv. Meteorol..

